# Evaluating the impact of HBM-based education on exercise among health care workers: the usage of mobile applications in Iran

**DOI:** 10.1186/s12889-020-08668-8

**Published:** 2020-04-22

**Authors:** Reza Jorvand, Fazlollah Ghofranipour, AliAsghar HaeriMehrizi, Mahmoud Tavousi

**Affiliations:** 1 Faculty of Health, Ilam University of Medical Sciences, Ilam, Iran; 2 Faculty of Medical Sciences, Tarbiat Modares University, Tehran, Iran; 3 Health Metrics Research Center, Iranian Institute for Health Sciences Research, ACECR, Tehran, Iran

**Keywords:** Early medical interventions, Exercise, Telegram messenger, Health personnel, Iran

## Abstract

**Background:**

Mechanical life made us witness the growing increase of chronic diseases despite the prominent scientific developments in the field of health, treatment and control. The aim of this study was to evaluate the impact of educational intervention based on Health Belief Model (HBM) using mobile applications (Telegram messenger) on doing exercise among the health care workers of Ilam university of medical sciences in 2017.

**Methods:**

In this interventional study, 114 people working in Ilam University of medical sciences participated in two groups of intervention and control (employed at two different cities) after providing the informed consent form. HBM-ISCS questionnaire was used to collect the required data and its reliability was approved using Cronbach’s alpha. Descriptive statistics, chi square, t test, repeated measures ANOVA (RMANOVA) and structural equation model (SEM) were used.

**Results:**

Half of the participants were men, 58.77% of them were undergraduate. The mean and SD of their age was 37.61 ± 4.88 years. Based on the results of the repeated measures analysis of variance test (before and after the intervention) in the intervention group, there were significant difference in all of the HBM constructs (perceived barriers was excepted), daily and weekly exercises, blood biochemical markers of the participants (*P* > 0.05). The above changes were not significant in the control group (*P* ≥ 0.05).

**Conclusion:**

Exercise is closely related to the beliefs of people, so implementing educational interventions based on Telegram messenger with emphasis on health beliefs and using HBM can lead to have exercise. Therefore, this application can be a suitable tool to deliver trainings, especially when holding in-person classes is difficult.

## Background

Today, with scientific advances in health and treatment, we are seeing an increasing increase in chronic diseases [1].

Chronic diseases are principally due to four health risk behaviors: physical inactivity, poor nutrition, tobacco use, and excess alcohol consumption [2].

In this regard, the importance of exercise is to such an extent that the World Health Organization (WHO) has identified it as the first indicator of health [3].

Inappropriate physical activity is one of the risk factors for chronic diseases [4] and is one of the leading causes of mortality worldwide which has doubled the risk of cardiovascular disease (CVDs)**)** [5, 6], and 23% of these deaths Causes of such diseases [7].

Low physical activity is one of the causes of overweight and obesity and obesity is a risk factor for non-communicable diseases such as hypertension and cardiovascular disease [8].

Studies have shown that exercising for 120–120 min at moderate intensity throughout the week can significantly reduce the risk factors for coronary artery disease [9], although in many countries physical activity levels are poor [10–12]. including Iran [13, 14].

Exercise is a form of organized physical activity that is regularly, repeatedly, and programmed with the aim of playing and having fun, gaining more fitness, health or fitness [15]. Its important role in preventing chronic diseases such as CVDs, diabetes, cancer and musculoskeletal diseases has been proven [16].

Promoting exercise is important because cardiovascular disease is the leading cause of death in Iran and the average age of Iranians with such diseases is 20 years younger than in the European countries and the USA [17].

Therefore, implementation of appropriate educational interventions based on health education models and theories is necessary.

One of the commonly used models in preventing diseases is health belief model (HBM), exclusively created for behaviors related to health [18]. This model has been the basis of many educational interventions to promote healthy behaviors including exercise [19].

HBM explains the quality of behavior change in relation to people’s health and helps educators evaluate and describe people’s health behaviors by understanding their beliefs about health [20]. Researchers believe that any kind of education leads to learning, but the depth and sustainability of learning varies in different ways [21, 22], and technology plays a major role in today’s knowledge sharing [23]. Technology-based learning is very useful in creating and developing geographically restricted learning environments and people are affected by information technology [23].

The use of modern educational technology is increasing so that in one study, both telegram and Instagram were used to assess the impact of continuing education on patients [24].

In spite of the above, few studies have examined the impact of using virtual networks and messaging programs in education in Iran, while these technologies are not limited to a specific time and place and have a favorable spread and influence among people.

Therefore, due to the increasing growth of telegram use and its features in Iran during this study, the research team decided to evaluate the impact of HBM-based educational interventions using telegram on exercise among health staff of Ilam University of Medical Sciences.

This article is part of a study. In the previous article extracted from the study referred to the psychometric section of the measurement instrument was reported [25] In the present article the relevant intervention section is reported.

## Methods

The present semi-experimental study was conducted in 2017 in the city of Ilam, Iran. Data were collected using a valid and reliable questionnaire (HBM-ISCS) [25] at two stages of before and 6 months after the educational intervention. The main purpose of the present study was to investigate the effect of HBM-based education on exercise as a CVD preventive factor.

In addition, blood biochemical markers (including FBS, TG, Cholesterol, HDL and LDL) were used as measurable markers to indicate changes due to the effect of training intervention on exercise.

### The conceptual framework

HBM is suitable for preventing chronic diseases when a researcher wants to educate people who are not sick. According to HBM, adopting a health behavior depends on people themselves believing in health problems (inactivity), accepting the reality, being sensitive to its impact on health, feeling threatened, listed as a problem. Take their health seriously and understand it. Different side effects (CVD) in different aspects of their health. Then, with guidance from their surroundings, they are convinced that preventive activities (such as doing exercise) are much less expensive than treating related diseases such as CVDs. As a result, they quickly take such precautionary measures. It is important that we understand why and how people adopt new behaviors, how they have changed behavior, and what has caused them. According to HBM, eople change their behavior when they realize that their illness is serious because they would otherwise become less healthy.

### The measure

Data were collected using a valid and reliable questionnaire (HBM-ISCS (at two stages of before and 6 months after the educational intervention.

The questionnaire includes 14 items based on the constructs of HBM (two items of perceived susceptibility, three items of perceived severity, two items of perceived benefits, three items of perceived barriers and four items of self-efficacy) to assess the health beliefs of health care workers about the impact of exercise on CVDs. The behavior contained two items for assessing daily and weekly exercise (in minutes). The validity and reliability of HBM-ISCS has been verified by the research team of the present study [25].

Cronbach’s alpha coefficients of 0.715 to 0.816 were calculated for the subscales, perceived susceptibility (0.725), perceived severity (0.715), perceived benefits (0.768), perceived barriers (0.727), self-efficacy (0.816) and HBM-ISCS (0.746).

### Sampling

The target population of this study consisted of health care workers in the healthcare networks of Ilam province. In order to prevent the delivery of provided information in educational interventions, the healthcare workers in two healthcare networks (draw from 8 health networks) with more than 120 km distance from each other were selected randomly, and by random allocation (by the draw method), the workers of one network were enrolled as intervention group and the workers of the other network were enrolled as control group.

**Fig. 1:**
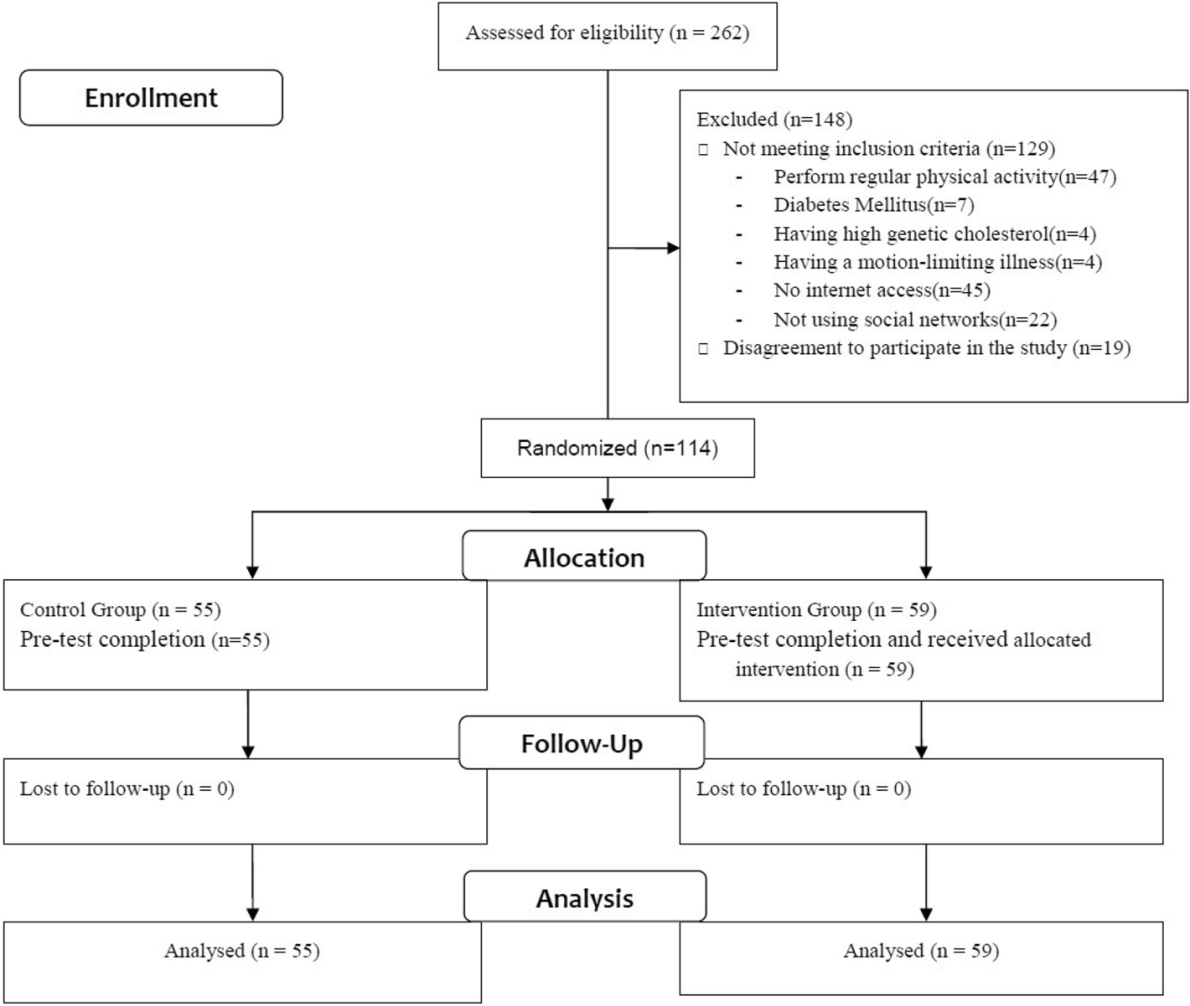
Flow diagram of participants through the study

First, two cities in Ilam Province were selected randomly and then a list of all Health Care Workers with inclusion terms was prepared by the administrative affairs department. The research team went to the workplace of health care workers and explained the study’s objectives and working methods and get a written informed consent from those applicants who wanted to participate in the research. In the following, all applicants were visited by a general physician and their health regarding CVDs was confirmed. At this stage, census method was employed to determine the sample size and for sampling, and all healthcare workers, who had no exercise (the criterion for not having exercise included not having at least 30 min of exercise frequently and with moderate severity for 4 days in a week), were listed to participate in the study if they agreed. It is to be noted that none of the participants in the interventional study was present in the descriptive section. Figure 1 displays the process of sampling, random allocation and follow-up.

#### Inclusion/exclusion criteria

Inclusion terms were having literacy, being formal or contractual employee, lack of developing chronic diseases or those leading to limit movement, not having daily exercise, having access to the internet and using social networks for education and completing informed consent form. Exclusion terms were resignation, dismissal, and failure to attend educational programs or developing a disease or condition limiting the movement.

#### Educational intervention

The educational intervention was carried out non-person and using the Telegram messaging application. First, a meeting was held with the participants to introduce the program and explaining the main issues of study (including the introduction of research team, objectives, schedule, expectations from the participants, how to communicate with research team, and alike). The non-person education program had two parts: sending the educational package via Telegram to the intervention group, and establishing a group in Telegram for all members of the intervention group. In this group, questions according to the content of educational package were provided while sending the package to provide the opportunity for discussion and interlocution of the group members. The duration of intervention was 2 weeks. The content of educational package was sent to the intervention group in three parts with the aim of providing a thinking opportunity for the participants and also using the reminding role of educational package (each part of the educational content was send every 2 weeks). The group members could send the pictures of their own exercise to the Telegram group. The follow-up duration in the present study was 6 months, and after completing the education course and also during the follow-up, members of the intervention group used to receive reminding messages through the Telegram group to encourage doing exercise.

**Fig. 2:**
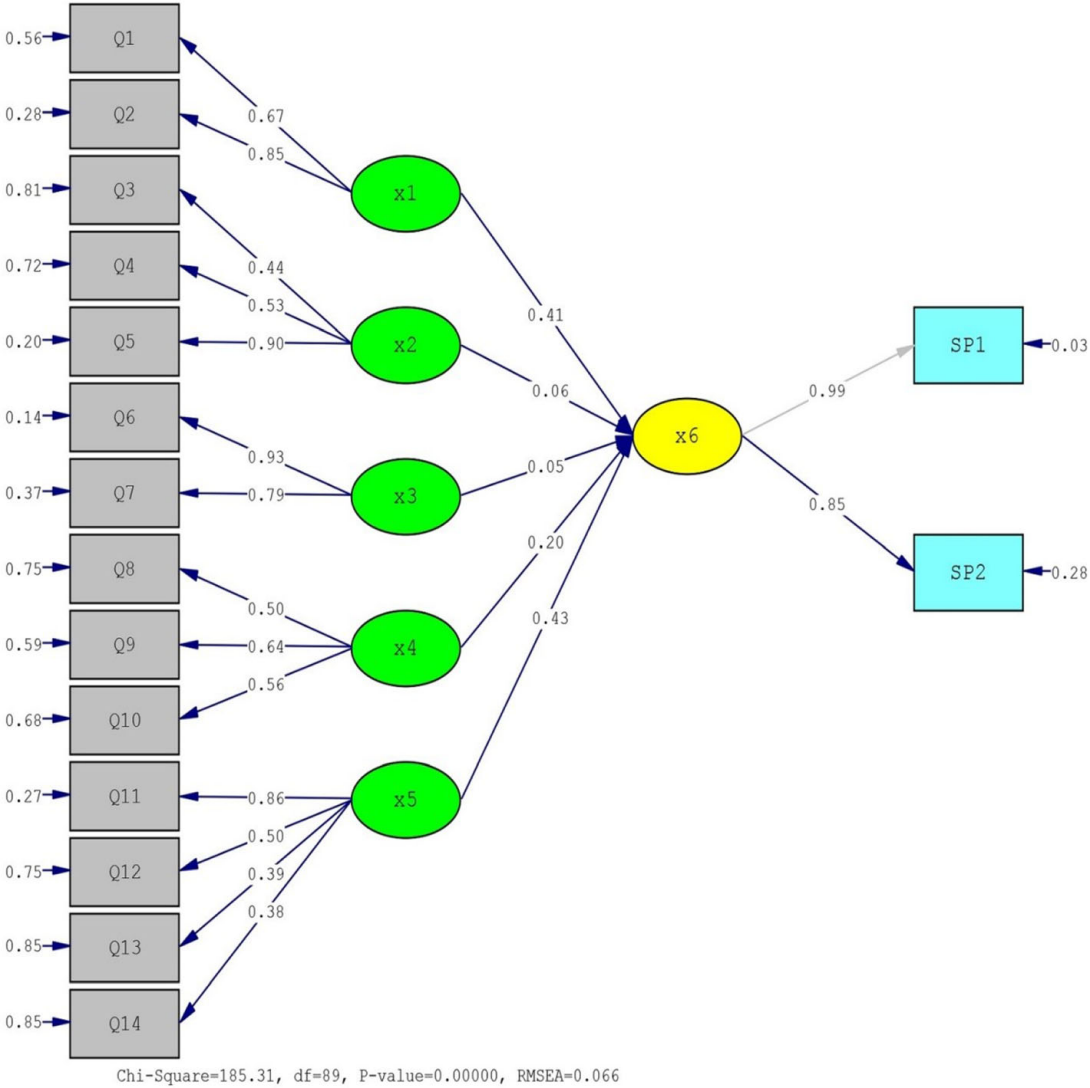
Structural Equation Model (SEM) Diagram

The content of educational intervention was designed based on descriptive study results, which was performed by the research group and it was shown that all constructs of HBM have the ability to predict the participants’ exercise behavior. The results of the descriptive study to evaluate the ability of HBM constructs to predict the participants’ exercise behavior using structural equation modeling (SEM) showed that the strongest predictive constructs were self-efficacy (43%), perceived susceptibility (41%) and perceived barriers (20%), orderly. The predictive power of the constructs of perceived severity and benefits was 6 and 5%, respectively (Table 1, Fig. 2).

**Table 1:** Fit indices in path analysis (SEM)

Fit indexes	
Degrees of Freedom (df)	89
χ^2^/ df	2.08
*P* value	0.001
Root Mean Square Error of Approximation (RMSEA)	0.066
Goodness of Fit Index (GFI)	0.91
Non-Normed Fit Index (NNFI)	0.94
Comparative Fit Index (CFI)	0.95
Root Mean Square Residual (RMR)	0.073
Standardized RMR (SRMR)	0.060
Incremental Fit Index (IFI)	0.95

Therefore, in addition to defining exercise, types of exercise, and other related topics, the training package included ways to increase self-efficacy, perceived susceptibility, and suggestions for reducing perceived barriers to daily exercise. The content was provided to intervention group only via Telegram and control group members did not receive any training course during the study.

In the present study, the female participants had access to the gym and aerobic exercise daily under the supervision of a sports instructor and the men had access to the gym for volleyball and futsal and mountaineering. Participants could at any time perform other sports such as hiking and running.

Controlling group exercise as well as its duration and severity was performed by the researcher or one of the colleagues on behalf of the researcher. However, due to the impossibility of controlling individual exercise, participants’ daily and weekly exercise data collection was ultimately self-reported. The information in this section was collected with three questions; the first question to examine daily exercise by the participants was answered yes or no. The second and third questions asked about the amount of exercise activities such as fast walking, running, football, futsal, volleyball, mountaineering and etc. in minutes and days per week.

The blood samples of the participants were taken in addition to completing the questionnaire at the two stages of before and 6 months after intervention. Some indices were measured such as fasting blood sugar (FBS), Cholesterol, Triglycerides (TGs), LDL and HDL, and were included in the study as objective evidence.

#### Data analysis

SPSS (ver. 16) was used to study data analysis. In addition to descriptive methods, we used chi-square, t-tests and repeated measures ANOVA (RMANOVA) in SPSS software for statistical analysis of data. Significance level in all measurements was considered *P* < 0.05.

LISREL (ver. 8.8) was used structural equation model (SEM).

## Results

Fifty percent of the participants were men and 58.77% of them had BSc degree. As indicated, there is no statistically significant difference between the two groups in terms of gender, age, marital status, education, work experience, daily and weekly exercise, in terms of family history of CVDs and death due to CVDs in the family (*P* ≥ 0.05).In other words, the two groups are homogenous (Tables 2 & 3).

**Table 2:** Survey of the groups’ consistency in terms of individual variables before intervention

Variables	Intervention group (*N* = 59)		Control group (*N* = 55)		*P*-value
	N	%	N	%	
Gender					
Female	29	49.2	28	50.9	0.500
Male	30	50.8	27	49.1	
Marriage					
Married	52	88.1	41	74	0.092
Single	7	11.9	14	26	
Education					
Diploma	8	13.6	6	10.9	
Associate Degree	12	20.3	9	16.3	0.516
Bachelor	31	52.5	36	65.4	
Master’s degree	8	13.6	4	7.2	
Exercise (daily)					
Yes	0	0	0	0	
No	59	100	55	100	
Variable	Mean ± SD		Mean ± SD		
Exercise (weekly)	13.3 ± 18.56		24.75 ± 20.18		0.627
Job experience	6.03 ± 13.8		6.03 ± 13.79		0.088
Age (years)	4.65 ± 37.64		6.14 ± 37.5		0.168

**Table 3:** CVD history among the participants

Variables	Intervention Group (*N* = 59)		Control Group (*N* = 55)		*P*-value
	N	%	N	%	
Family history of CVDs					
Yes	21	35.6	25	45.4	0.519
No	33	55.9	25	45.4	
I do not know	5	8.5	5	9.2	
The history of death from CVDs in the family					
Yes	6	10.2	8	14.5	0.404
No	52	88.1	44	80	
I do not know	1	1.7	3	5.5	

Based on the results of the repeated measures analysis of variance test for HBM structures in intervention group, there was no significant difference in perceived barriers (before and after the intervention) (*P* ≥ 0.05); but in other structures, this difference was significant (*P* > 0.05). The above changes were not significant for all constructs in the control group (P ≥ 0.05). Given the significance of the interaction between time and group in perceived susceptibility, the results of the main effects test may be misleading. Therefore, t-test (Bonferroni correction) was used to compare groups at different times. There was no significant difference between groups before intervention for perceived susceptibility (*P* = 0.154), but there was a significant difference after intervention (*P* = 0.036) (Table 4).

**Table 4:** Comparison of HBM structures between the intervention and control groups before and after intervention by RMANOVA

Variables	Pre-intervention	Post-intervention	*P*-value	F
	Mean ± SD	Mean ± SD		
Perceived susceptibility^a^				
Intervention Group	0.81 ± 9.57	0.18 ± 9.97	Sig. = 0.018	F(1,112) = 5.718
Control Group	0.77 ± 9.74	0.54 ± 9.76		
Perceived severity				
Intervention Group	1.02 ± 13.85	1.82 ± 12.78	Sig. = 0.000	F(1,112) = 20.390
Control Group	1.77 ± 12.93	1.9 ± 13.14		
Perceived benefits				
Intervention Group	0.58 ± 9.63	1.06 ± 10.9	Sig. = 0.010	F(1,112) = 6.913
Control Group	1.13 ± 8.89	1.36 ± 9.03		
Perceived barriers				
Intervention Group	3.01 ± 9.46	2.22 ± 8.24	Sig. = 0.634	F(1,112) = 0.998
Control Group	3.21 ± 8.71	3.12 ± 9.11		
Self-efficacy				
Intervention Group	2.99 ± 16.78	1.32 ± 19.18	Sig. = 0.024	F(1,112) = 0.955
Control Group	3.16 ± 16.21	2.94 ± 16.41		

^a^ The interaction between time and group was significant

Based on the results of the repeated measures analysis of variance test, there was significant difference between daily and weekly exercise before and after the intervention in intervention group (*P* > 0.05). The above changes were not significant in the control group (*P* ≥ 0.05).

Given the significance of the interaction between time and group in daily exercise and weakly exercise, the results of the main effects test may be misleading. Therefore, t-test (Bonferroni correction) was used to compare groups at different times. There were no significant difference between groups before intervention for daily exercise and weakly exercise (*P* ≥ 0.05), but there were a significant difference after intervention in both them (*P* > 0.05) (Table 5).

**Table 5:** Comparison of the average regular activity between the intervention and control groups before and after intervention by RMANOVA

Variables	Pre-intervention	Post-intervention	*P*-value	F
	Mean ± SD	Mean ± SD		
Daily exercise (Minute)^b^				
Intervention Group	0^a^	1.72 ± 25.23	Sig. = 0.001	F(1,112) = 105.010
Control Group	0^a^	4.16 ± 1.09		
Weakly exercise (Minute)				
Intervention Group	31.34 ± 18.55	41.74 ± 129.76	Sig. = 0.001	F(1,112) = 94.182
Control Group	24.75 ± 20.18	3.85 ± 24.54		

^a^The groups without daily exercise

^b^The interaction between time and group was significant

Based on the results of the repeated measures analysis of variance test for blood biochemical markers of the participants in intervention group, there were no significant difference in TG, Cholesterol, LDL and HDL (before and after the intervention) (*P* ≥ 0.05); but in FBS, this difference was significant (*P* > 0.05).

Given the significance of the interaction between time and group in FBS, the results of the main effects test may be misleading. Therefore, t-test (Bonferroni correction) was used to compare groups at different times. There was no significant difference between groups before intervention for FBS (*P* = 0.604), but there was a significant difference after intervention (*P* = 0.003) (Table 6).

**Table 6:** Comparison of the mean blood indices between the intervention and control groups before and after intervention- by RMANOVA

Variables	Pre-intervention	Post-intervention	*P*-value	F
	Mean ± SD	Mean ± SD		
FBS (Mg/dl)				
Intervention Group	7.92 ± 77.91	7.19 ± 76.23	Sig. = 0.771	F(1,112) = 0.085
Control Group	8.72 ± 78.72	9.03 ± 80.94		
TG (Mg/dl)				
Intervention Group	24.83 ± 166.01	23.41 ± 160.29	Sig. = 0.732	F(1,112) = 0.118
Control Group	27.46 ± 164.65	25.41 ± 171.02		
Cholesterol (Mg/dl)				
Intervention Group	30.16 ± 185.02	26.96 ± 181.15	Sig. = 0.822	F(1,112) = 0.051
Control Group	29.04 ± 186.32	28.25 ± 192.09		
LDL (Mg/dl)				
Intervention Group	21.78 ± 115	20.07 ± 112.25	Sig. = 0.982	F(1,112) = 0.0001
Control Group	21.01 ± 116.16	21.30 ± 117.65		
HDL (Mg/dl)				
Intervention Group	6.06 ± 37.01	5.26 ± 38.56	Sig. = 0.872	F(1,112) = 0.026
Control Group	5.86 ± 37.25	3.79 ± 35.95		

## Discussion

The present research results showed that before the educational intervention, there was no significant difference in the mean of HBM constructs and also in the amount of the participants’ daily and weekly physical activities, but the educational intervention using Telegram led to significant changes in the HBM constructs, and consequently, the participants’ daily and weekly exercise.

Perceived susceptibility is one of the very effective factors in adopting preventive behaviors [26, 27]. In the study by Zeinali conducted on the evaluation of impact of educational intervention on promoting preventive behaviors for CVDs among people with natural Angiography [26], and also in the study by Amirzade, the educational intervention led to the change of mean score of perceived susceptibility of women that is consistent with the results of present study [28]. In the study by Karimi, there was a significant change of all HBM constructs, including perceived susceptibility before educational intervention [29]. In this study, the increase of perceived susceptibility was directly related to the increase of preventive behavior (i.e. doing exercise) for CVDs.

Not having sufficient understanding about the seriousness of CVDs risk (i.e. low perceived severity) can be a barrier in the way of changing the lifestyle of people and preventing such diseases [30]. In the present study, we observed the significant increase of the mean scores of perceived severity in the intervention group after intervention, which can be one of the reasons of behavior change among the participants about exercise; this result is supported by various studies. For instance, in the study of Tahernia, the mean scores of perceived severity had significant increase after intervention with the aim of promoting preventive behaviors for CVDs [31]. Also Mardani and Shamsi observed significant changes of HBM constructs after intervention that are same as the present study [32, 33] .

Individual preventive behavior is defined as the perception of an individual about the benefits and barriers of behaviors and balance between them [34]. On the other hand, the more is the perceived benefits of adopting preventive behavior in a person’s opinion, the greater will be the possibility of doing that behavior. In a study by Abood, the educational intervention led to increasing the perceived benefits effectively in the intervention group [35], and in the study of Ghaderian, which was conducted with the aim of evaluating the impact of educational intervention on promoting preventive behaviors for CVDs among health care workers in the health centers of the west of Ahvaz city, Iran, there was a significant increase in the scores of perceived benefits [36]. On the other hand, one of the defining factors of doing exercise is the barriers a person encounters with while taking these behaviors, and in contrast, his/her ability to cope with the barriers of doing physical activity is significantly and positively related to the increase of exercise [37, 38], to the extent that some researchers have reported that perceived barriers are among the most important component of HBM in conducting recommended behaviors [39]. The importance of perceived barriers and benefits is especially observed when the goal of suggested behavior is to prevent an unhealthy behavior [40].

In the present study, the educational intervention led to the significant decrease of perceived barriers in the intervention group for doing exercise, and the reverse relationship between the scores of perceived barriers and exercise was observed. In the study of Peyman with the aim of evaluating the impact of educational intervention on the lifestyle of girl students, there was a significant decrease of perceived barriers among the participants about exercise and healthy diet [41]. Also the findings of Mohseni-pouya support the results of this study, implying educational intervention significantly decreases barriers of people’s perception about preventing CVDs [42].

In this study, the mean score of perceived barriers was decreased significantly as one of the predictive factors for exercise behavior after intervention. This decrease, besides the increase of perceived benefits, highlighted the benefits of exercise among the participants in the intervention group and increased the amount of daily and weekly exercise significantly in this group.

Self-efficacy is among those variables that results in the increase of exercise in people [43] and among the effective factors for taking healthy behaviors [44]. The results of the present study showed that there is a direct relationship between self-efficacy and exercise, which is consistent with the results of other studies [45–47]. Also the increase of self-efficacy after educational intervention could increase the mean amount of the participants’ daily and weekly exercise.

Our results showed that doing daily exercise and the mean of daily and weekly exercise were also increased in the control group though this increase was not significant. We talked with three members of the control group who began daily exercise and found out that two of them according to the suggestion of their physician and one because of concerns about overweight have begun daily exercise in a limited amount. These results are consistent with the results of other studies about exercise. For example, the educational intervention in the study of Tahernia using HBM led to a significant statistical difference in the physical activity amount of the participants [31]; this result is in agreement consistent with findings of Hadad in Jordan [48]. Furthermore, various other studies have reported a significant increase in the amount of physical activity after educational intervention [49–53].

In simple terms, educational intervention for doing exercise resulted in controlling FBS, TG, Cholesterol and LDL in the intervention group and also decreased the mean level of blood biochemical markers between 1.67 and 5.72 units, while the lack of exercise in the control group led to the increase of the mean level of these indices for about 1.49 to 6.36 units. In other words, exercise could improve the mean of blood biochemical markers in the intervention group and not increased or worsened the mean of blood biochemical markers in the control group. This shows that though exercise could not make any significant changes in the intervention group but led to control and minor the improvement of these indices, which is very important. Meanwhile, the mean of HDL for the intervention group members was increased by as much as 1.54 units post-intervention; in other words, exercise resulted in improving the blood level of HDL in the intervention group though this change was not significant. On the other hand, the mean of HDL in the control group decreased by 1.31 units; in other words, not having exercise worsened the HDL level in the control group. As mentioned above, intervention based on exercise could not make any significant changes in the blood biochemical markers of the intervention group, and the research team tried to explain its reason(s). Thus, it was tried to identify similar studies and compare their results with the results of the present study; for example, in the study of Sardar and colleagues [54], 8 weeks of aerobic exercise led to decrease the FBS, Cholesterol, LDL and HDL but the changes were not significant statistically. Ramalho [55] and Herbst [56] also observed similar results to the findings of Sardar [54].

Alizadeh et al. also studied the impact of 40-min walking with moderate severity for 5 days a week in three groups (40 min continuously, 40 min periodic without physical activity); the results showed that these three groups had no significant difference in changes of Cholesterol, FBS and TG statistically [57]. An important point in Alizadeh’s study was that all the three groups had limited daily calorie and received their dietary advice from a dietitian [57].

The results of all of the above studies are consistent with the present study. These studies have shown that exercise alone is not sufficient to make a significant change in the blood biochemical markers and requires reviewing the intervention program. This review can be done on the amount of exercise, its severity, follow-up period or addition of other sections such as diet to the intervention. This has been confirmed in numerous studies where the interventions were based on lifestyle or combination of healthy diet and physical activity, which were done on people from different age groups and in different countries around the world with different cultures; in these studies, the interventions led to improve the levels of FBS, TG, Cholesterol, LDL and HDL significantly [58–62].

The results of this section of the study, assuming a same diet among the study groups, found that exercise can control and make minor improvement in the mean of FBS, TG, Cholesterol and HDL in the intervening group, and the lack of exercise worsens their level in the control group; this shows the impact of exercise in controlling the blood indices. Even though the intervention based on exercise could not make any significant changes in the blood indices of the intervention group, it led to decrease/control the blood indices in them, which is of high importance itself. Because, for example, 1 % decrease in the amount of Cholesterol and LDL can decrease the amount of risk factors of CVDs accordingly [63].

### Limitations

Among the limitations of this study, we can refer to the impact of some factors including personality traits and psychic moods when answering the questionnaire as well as the inability to control the dietary status of the participants. Extracting information based on self-reporting is also among the shortcomings of this study.

## Conclusions

The results of this study indicated that exercise is closely related to the health care worker’s beliefs, and applying HBM can be well effective in this regard. Therefore, implementing educational interventions with emphasis on health beliefs and using HBM can be an appropriate strategy to change health care worker’s behavior (doing exercise) in order to prevent CVDs. In addition, exercise is effective in controlling the level of FBS and stopping its retardation towards an undesirable level of health though this variable alone is not enough to improve the above indices.

The results also showed that Telegram is a perfect tool to deliver educational materials, especially in the difficult conditions of holding in-person classes. Therefore, despite the lack of access to this application in Iran in the current situation, the results of the present study showed that modern messaging technologies in the form of messengers, media and social networks can be used to deliver information and, in some cases, the desired health skills in order to achieve the ultimate goal of promoting the health care workers’ health level and quality of life.

## Data Availability

All data generated and/or analyzed during the current study are available by the responsible author if needed.

## Abbreviations

HBM: Health Belief Model
CVDs: Cardiovascular diseases
SEM: Structural Equation Modeling
ISCS: Impact of Sport on the Cardiovascular Diseases Scale

## References

[1] Kamran A, Heydari H (2015). Predictive Power of the Trans Theoretical Model for Physical Activity in Patients with Diabetic Patients. J Health.

[2] Milani RV, Bober RM, Lavie CJ (2016). The role of Technology in Chronic Disease Care. Prop Cardiovascular Dis.

[3] Rahmati-Najarkolaei F, Ghaffarpasand E, Gholami Fesharaki M (2015). Nutrition and physical activity educational intervention on CHD risk factors: a systematic review study. Arch Iranian Med.

[4] Zarenezhad M, Farshidi H, Zare S (2012). Investigating the awareness of inter-city bus drivers and truck drivers on coronary heart diseases risk factors. Hormozgan Med J.

[5] Zare F, Aghamolaei T, GHanbarnejad A (2016). The effect of Transtheoretical model-based education on promoting physical activity among employees of Abumusa Island. Iran J Health Syst Res.

[6] Rejali M, Mostajeran M. Assessment of physical activity in medical and public health students. J Education Health Promotion. 2013;2:19. 10.4103/2277-9531.112690 PMC3778645 24083269

[7] Ghahramanian A, Heidarzadeh M (2011). Rostami H et al. Behavioral risk factors for cardiovascular diseases in Bonab city employees Journal of Holistic Nursing And Midwifery.

[8] Cooper R, Mishra GD, Kuh D (2011). Physical activity across adulthood and physical performance in midlife: findings from a British birth cohort. Am J Prev Med.

[9] Tehrani H, GHolian AM, Hasani KM (2016). The impact of new communications technology on promoting women’s physical activity. Payesh.

[10] Zimmermann-Sloutskis D, Wanner M, Zimmermann E (2010). Physical activity levels and determinants of change in young adults: a longitudinal panel study. Int J Behavioral Nutrition Physical Activity.

[11] Brochado A, Oliveira-Brochado F, Brito PQ (2010). Effects of personal, social and environmental factors on physical activity behavior among adults. Revista Portuguesa de Saúde Pública.

[12] Ishii K, Shibata A, Oka K (2010). Environmental, psychological, and social influences on physical activity among Japanese adults: structural equation modeling analysis. Int J Behavioral Nutrition Physical Activity.

[13] Moeini B, Rahimi M, Hazaveie S (2010). Effect of education based on trans-theoretical model on promoting physical activity and increasing physical work capacity. J Mil Med.

[14] Momenan A, Delshad M, Mirmiran P (2012). Physical inactivity and related factors in an adult Tehranian population (Tehran Lipid and Glucose Study). Iran J Endocrinology Metabolism.

[15] A MS (2016). Designing and evaluating physical education curriculum in female students. Tarbiat Modares University.

[16] Dishman R, Heath G, Lee I-M (2013). Physical activity epidemiology 2nd edition. Human Kinetics.

[17] Akbari Z, Mohammadi M, Effati B (2016). The Survey on the Prevalence of the Cardiovascular Diseases Risk Factors among the Qom University of Medical Sciences Staffs in 2012 Paramedical Sciences and Military Health.

[18] Saffari M, S D (2008). Principles and foundations of health promotion and education.

[19] Hatefnia E, Ghazivakili Z (2016). Factors Associated with Regular Physical Activity for the Prevention of Osteoporosis in Female Employees Alborz University of Medical Sciences: Application of Health Belief Model Alborz University. Med J.

[20] Glanz KA, Rimer BA, K. (2008). V. Health behavior and health education theory, research and practice.

[21] Vadeboncoeur T, Bobrow BJ, Clark L (2007). The save hearts in Arizona registry and education (SHARE) program: who is performing CPR and where are they doing it?. Resuscitation.

[22] Taylor AM (1941). The Workshop. Am J Nursing.

[23] Anbari A, Hariri N (2015). The role of Persian professional web-based social networks in knowledge sharing. Nastinfo.

[24] Farsi Z (2016). Designing and Implementation of Evaluation Checklist for Student Educational Performance. MCS.

[25] Jorvand R, Tavousi M, Ghofranipour F. Impact of Sport on the Cardiovascular Diseases Scale Based on Health Belief Model: Questionnaire Psychometric Properties. Iran Red Crescent Med J. 2018.

[26] Zainali M, Asadpour M, Aghamolaei T (2015). Effect of educational intervention based on health belief model to promote preventive behaviors of cardiovascular disease in people with normal angiographic results. J Prev Med.

[27] Tavasoli E, Hassanzadeh A, Ghyasvand R (2010). The impact of education on the health belief model to promote preventive nutritional habits of heart disease-disease of the housewives. J School Health Institute Health Res.

[28] Amirzadeh Iranagh J, Motallebi S (2016). The effect of health belief model based on education intervention on physical activity of elderly women. J Urmia Nursing Midwifery Faculty.

[29] Karimy S, Mansouri A, SHahdadi H (2016). The effect of health belief model-based education on adherence to the dietary regimen in pregnant women with gestational diabetes. J Diabetes Nurs.

[30] Sadler M (2005). Soy and health 2004: clinical evidence, dietetic applications. Nutr Bull.

[31] Taheriniya A, Ebrahimpuriyan L (2013). Mohsenzadeh Y. Effect of education on knowledge and performance of activity in patients with myocardial infarction: Health Belief Model Alborz University Medical Journal.

[32] Piri A (2010). Mardani Hamuleh M. A SV Effects of education based on health belief model on dietary adherence in diabetic patients Journal of Diabetes and Metabolic Disorders.

[33] Shamsi M, Bayati A, Mohamadbeygi A (2010). The effect of educational program based on health belief model (HBM) on preventive behavior of self-medication in woman with pregnancy in Arak. Iran Pejouhandeh.

[34] Morowatisharifabad M, Rouhani Tonekaboni N (2007). The relationship between perceived benefits/barriers of self-care behaviors and self management in diabetic patients. J Hayat.

[35] Abood DA, Black DR, Feral D (2003). Nutrition education worksite intervention for university staff: application of the health belief model. J Nutr Education Behav.

[36] G M (2015). The Effect of Education Based on Health Belief Model on Promoting Preventive Behaviors of Cardiovascular Diseases in Health Center Personnel in Ahwaz, 2014.

[37] Sallis JF, Prochaska JJ, Taylor WC (2000). A review of correlates of physical activity of children and adolescents. Med Sci Sports Exercise.

[38] Lee L-L, Arthur A, Avis M (2008). Using self-efficacy theory to develop interventions that help older people overcome psychological barriers to physical activity: a discussion paper. Int J Nursing Studies.

[39] C FG (2005). William's Obstetrics.

[40] Gs Z (2014). Designing and evaluating a theory-based educational intervention for changing physical activity in spouses of staff, Health Education.

[41] Peyman N, Mahdizadeh M, Mahdizadeh M (2014). Evaluation of education in promoting healthy lifestyle behaviors among adolescent girls, according to the Health Belief Model. JSUMS.

[42] Mohsenipouya H, Esmaeili Shahmirzadi S, Seifi Makerani A (2017). Efficacy of educational intervention about the Prevention of Cardiovascular Disease among Adolescent Boys; an application. Health Belief Model Tolooebehdasht.

[43] Foley L, Prapavessis H, Maddison R (2008). Predicting physical activity intention and behavior in school-age children Pediatric Exercise. Science.

[44] Kessler TA. Increasing mammography and cervical cancer knowledge and screening behaviors with an educational program. Oncol Nurs Forum. 2012:61–8. 10.1188/12.ONF.61-68 22201656

[45] Kirk A, MacMillan F, Webster N (2010). Application of the transtheoretical model to physical activity in older adults with type 2 diabetes and/or cardiovascular disease. Psychol Sport Exerc.

[46] Jalilian F, Emdadi S, Mirzaie M (2011). The survey physical activity status of employed women in Hamadan University of Medical Sciences: the relationship between the benefits, barriers, self-efficacy and stages of change. J Yazd Health College.

[47] Aghamolaei T, Tavafian SS, H L (2009). Exercise self-efficacy, exercise perceived benefits and barriers among students in Hormozgan University of Medical Sciences. Iran J Epidemiology.

[48] Al-Ali N, Haddad LG (2004). The effect of the health belief model in explaining exercise participation among Jordanian myocardial infarction patients. J Transcultural Nursing.

[49] Dorairaj P, Panniyammakal J, Shifalika G (2009). Impact of a worksite intervention program on cardiovascular risk factors: a demonstration project in an Indian industrial population. J Am College Cardiology.

[50] Villablanca AC, Arline S, Lewis J (2009). Outcomes of national community organization cardiovascular prevention programs for high-risk women. J Cardiovascular Translational Res.

[51] Lieber SB, Redberg RF, Blumenthal RS (2012). A national interactive web-based physical activity intervention in women, evaluation of the American Heart Association choose to move program 2006–2007. Am J Cardiology.

[52] Eshah NF, Bond AE, Froelicher ES (2010). The effects of a cardiovascular disease prevention program on knowledge and adoption of a heart healthy lifestyle in Jordanian working adults. Eur J Cardiovasc Nursing.

[53] Harting J, van Assema P, van Limpt P (2006). Cardiovascular prevention in the Hartslag Limburg project: effects of a high-risk approach on behavioral risk factors in a general practice population. Prev Med.

[54] Sardar M, Gaeini A, Ramezani J (2008). The effect of 8-weeks of regular physical activity on blood glucose, body mass index, maximal oxygen uptake (Vo2max) and risk factors cardiovascular diseases in patients with type of 1 diabetes mellitus Iranian journal of. Endocrinol Metab.

[55] Ramalho AC, de Lourdes LM, Nunes F (2006). The effect of resistance versus aerobic training on metabolic control in patients with type-1 diabetes mellitus. Diabetes Res Clin Pract.

[56] Herbst A, Bachran R, Kapellen T (2006). Effects of regular physical activity on control of glycemia in pediatric patients with type 1 diabetes mellitus. Arch Pediatrics Adolescent Med.

[57] Alizadeh Z, Kordi R, Hossein-Zadeh Attar MJ (2011). The effects of continuous and intermittent aerobic exercise on lipid profile and fasting blood sugar in women with a body mass index more than 25 kg/m2: A randomized controlled trial Tehran University Medical. J TUMS Publications.

[58] Davis JN, Ventura EE, Tung A (2012). Effects of a randomized maintenance intervention on adiposity and metabolic risk factors in overweight minority adolescents. Pediatric Obesity.

[59] Kuller LH, Gabriel KKP, Kinzel LS (2012). The women on the move through activity and nutrition (WOMAN) study: final 48-month results. Obesity.

[60] Vos RC, Wit JM, Pijl H (2011). The effect of family-based multidisciplinary cognitive behavioral treatment in children with obesity: study protocol for a randomized controlled trial. Trials.

[61] Christian JG, Byers TE, Christian KK (2011). A computer support program that helps clinicians provide patients with metabolic syndrome tailored counseling to promote weight loss. J Am Dietetic Assoc.

[62] Giardina E-GV, Sciacca RR, Foody JM (2011). The DHHS Office on Women's Health Initiative to Improve Women's Heart Health: focus on knowledge and awareness among women with cardiometabolic risk factors. J Women's Health.

[63] National CEPN (2002). Third report of the National Cholesterol Education Program (NCEP) expert panel on detection, evaluation, and treatment of high blood cholesterol in adults (Adult Treatment Panel III) final report Circulation.

